# Cold Plasma-Induced Changes in *Stevia rebaudiana* Morphometric and Biochemical Parameter Correlations

**DOI:** 10.3390/plants12081585

**Published:** 2023-04-08

**Authors:** Augustė Judickaitė, Justinas Venckus, Kazunori Koga, Masaharu Shiratani, Vida Mildažienė, Rasa Žūkienė

**Affiliations:** 1 Faculty of Natural Sciences, Vytautas Magnus University, Kaunas 44248, Lithuania; 2 Faculty of Information Science and Electrical Engineering, Kyushu University, Fukuoka 819-0395, Japan; 3 Center for Novel Science Initiatives, National Institutes of Natural Sciences, Tokyo 105-0001, Japan

**Keywords:** *Stevia rebaudiana* Bertoni, cold plasma, secondary metabolites, steviol glycosides, morphometric parameters

## Abstract

*Stevia rebaudiana* Bertoni is an economically important source of natural low-calorie sweeteners, steviol glycosides (SGs), with stevioside (Stev) and rebaudioside A (RebA) being the most abundant. Pre-sowing seed treatment with cold plasma (CP) was shown to stimulate SGs biosynthesis/accumulation up to several fold. This study aimed to evaluate the possibility to predict CP-induced biochemical changes in plants from morphometric parameters. Principle component analysis (PCA) was applied to two different sets of data: morphometric parameters *versus* SGs concentrations and ratio, and morphometric parameters *versus* other secondary metabolites (total phenolic content (TPC), total flavonoid content (TFC)) and antioxidant activity (AA). Seeds were treated for 2, 5 and 7 min with CP (CP2, CP5 and CP7 groups) before sowing. CP treatment stimulated SGs production. CP5 induced the highest increase of RebA, Stev and RebA+Stev concentrations (2.5-, 1.6-, and 1.8-fold, respectively). CP did not affect TPC, TFC or AA and had a duration-dependent tendency to decrease leaf dry mass and plant height. The correlation analysis of individual plant traits revealed that at least one morphometric parameter negatively correlates with Stev orRebA+Stev concentration after CP treatment.

## 1. Introduction

**Figure 1:**
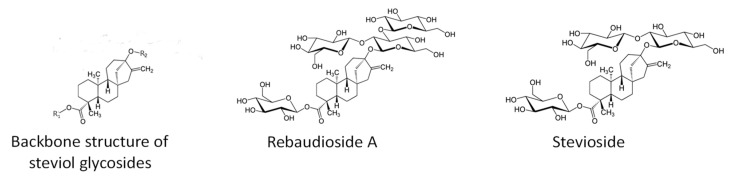
Backbone structure of steviol glycoside and chemical formulas of rebaudioside A and stevioside.

*Stevia rebaudiana* Bert. (Bertoni) is a perennial shrub indigenous to Paraguay, South America, which is now cultivated worldwide for its commercially important sweet-taste secondary metabolites steviol glycosides (SGs) [1]. To date, more than 40 different steviol glycosides have been described [2], which are diterpenes based on the ent-kaur-16-en-19-oic acid backbone structure with different bound sugar moieties (Figure 1). The most abundant SGs in *S. rebaudiana* are rebaudioside A (RebA) and stevioside (Stev)—these two substances may account for more than 90% of the total SGs found in dry stevia leaves. They are used in the food industry as natural non-caloric sweeteners since they are up to 400 times sweeter than sucrose [3]. As compared to Stev, RebA has an additional glucose monomer that gives it a higher sweetening potency and better quality of taste. Therefore, a higher RebA/Stev ratio and RebA concentration are preferred in stevia products.

SGs are gaining popularity as sweeteners due to their confirmed health benefits in vivo and in vitro. Besides the sweet taste, stevia extract and SGs demonstrate therapeutic benefits as a joint nutraceutical in the management of chronic diseases including overweight and obesity, diabetes mellitus, fatty liver, cardiac fibrosis, liver fibrosis, inflammatory bowel disease, certain types of cancer, hypertension, and chronic kidney diseases (reviewed in [4]), and they exhibit neuroprotective and antiepileptic activity [5].

Because of continuously increasing consumer and industrial demand, numerous efforts have been made to improve SG yield by manipulating physical and chemical factors during cultivation or by selecting plant genetic traits. The genetic gain in stevia is restricted due to various breeding challenges, including poor seed germination, cross-pollination, self-incompatibility, insufficient access to wild germplasm [6], and non-availability of trait-specific functionally relevant genomic resources for marker-assisted breeding [7]. Other methods and their combinations explored to increase SG yield are optimisation of cultivation conditions (photoperiod length, moisture, temperature) [8], fertilisation including biofertilisers [9], treatment with nanoparticles [10,11], modification post-harvest procedures such as drying, extraction or purification, and enzymatic conversion of Stev to RebA [12,13,14]. Most of these methods are often expensive or polluting. In addition, field growing conditions that are climate-dependent cannot be easily modified.

Pre-sowing seed processing with physical or chemical stressors is one of the techniques used not only to ameliorate germination and seedling growth [15,16] but also to improve other plant agronomic traits, and to increase disease resistance and concentrations of valuable biomolecules. Seed treatment with CP is regarded as an environmentally friendly method used to improve various plant properties including the synthesis of secondary metabolites [16,17]; therefore, the scientific field of plasma agriculture is gaining increasing attention this decade. CP is a non-equilibrium gas discharge plasma, consisting of charged particles, such as ions, free electrons and neutral particles, including gas molecules, free radicals and also UV photons. In CP, the particles are not in thermodynamic equilibrium, and the electron temperature in a plasma can be several orders of magnitude higher than the temperature of the neutral species or the ions, which is near room temperature [18].

Although CP treatment effects on various morphometric and biochemical plant traits were assessed for numerous plant species [19,20,21,22,23,24,25,26,27,28], CP has not been investigated on stevia until recently. To date, there is a single report of our group demonstrating a stimulating effect of low-pressure capacitively coupled plasma on SG biosynthesis in *Stevia rebaudiana* cv. ‘Criolla’ [29]. Since there is a lack of information on CP-induced morphometric changes and their relationship with secondary metabolites content in stevia, this study aimed to evaluate the possibility to predict biochemical changes induced by pre-sowing seed treatment with atmospheric pressure dielectric barrier discharge (DBD) CP from morphometric plant parameters. Principle component analysis (PCA) was applied on two different sets of data: morphometric parameters compared to the SG concentrations and ratio, and morphometric parameters compared to the other secondary metabolites (total phenolic content (TPC), total flavonoid content (TFC)) and antioxidant activity (AA).

## 2. Results

### 2.1. Effects on Germination

**Figure 2:**
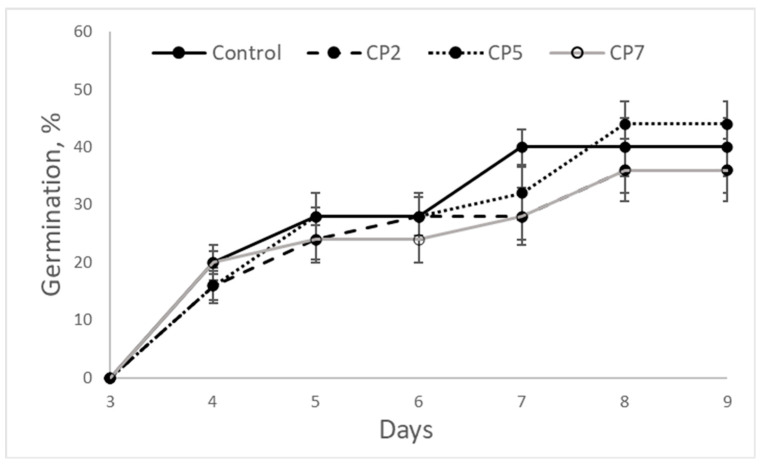
Germination kinetics of *Stevia rebaudiana* seeds (mean ± SEM, n = 4).

Based on our previous study of the stimulating effect of low-pressure CP on stevia cv. ‘Criolla’ seeds [29], the chosen duration for seed treatment was 2, 5 and 7 min, these treatments are further abbreviated as CP2, CP5 and CP7, respectively. The seeds were sown on the 6th day after CP treatment and the cumulative number of germinated seeds was counted each day. The germination results are presented in Figure 2. The germination curves for control and treatment groups are overlapping and do not show remarkable changes induced by CP. Richards plots [30,31] were used to determine the main indices of germination kinetics for quantitation of differences, and data are presented in Table 1. The treatments did not change the germination percentage (Vi), nor median germination time (Me) or germination rate. All CP treatments increased the value of quartile deviation (Qu) indicating bigger dispersion of germination time (less uniform germination).

### 2.2. Effects on Morphometric Parameters

Pre-sowing seed treatment with CP did not affect 12-week-old plant height, number of leaves or leaf dry mass per plant (Table 2). CP has a general duration-dependent tendency to decrease leaf dry mass and plant height.

### 2.3. Effects on Concentrations of Steviol Glycosides

**Figure 3:**
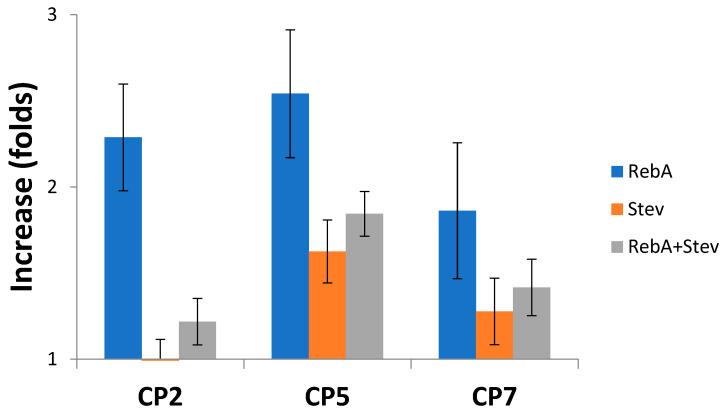
CP-induced changes in concentration of RebA, Stev and total concentration of RebA and Stev (RebA+Stev) in stevia leaves compared to control. Means ± SEM are presented (n = 7).

All seed treatments considerably increased RebA and RebA+Stev concentrations in leaves as compared to the control (Table 3). The increase in fold compared to the control is shown in Figure 3. CP treatments increased RebA concentration from 1.9- to 2.5-fold. CP5 induced the highest increase of RebA, Stev and RebA+Stev concentrations (2.5-, 1.6-, and 1.8-fold, respectively); however, the ratio of RebA/Stev was statistically increased only in the CP2 group.

### 2.4. Effects on Total Phenolic Content, Flavonoid Content and Antioxidant Activity

In contrast to CP-induced stimulation of SGs production, these treatments did not affect the content of total phenolic content (TPC), flavonoid content (TFC) or antioxidant activity (AA) (Table 4).

The effect of CP treatment on antioxidant activity in stevia leaves was evaluated by measuring the scavenging of the stable 2,2-diphenyl-1-picrylhydrazyl free radical (DPPH) and the results are shown in Table 4. CP treatment did not induce statistically significant changes in AA.

### 2.5. Principle Component and Correlation Analysis

**Figure 4:**
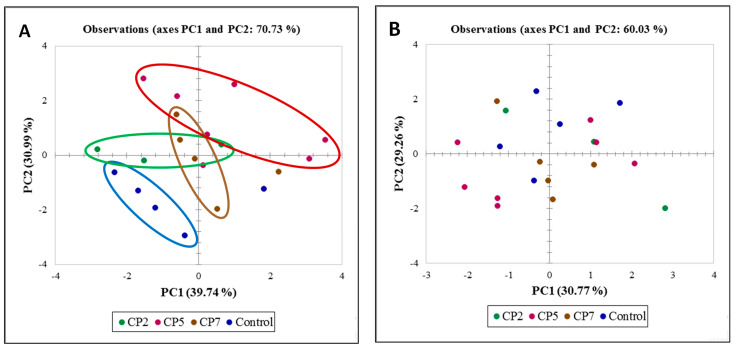
Biplot of principle components of the control group and treatment groups (CP2, CP5 and CP7) based on (**A**) morphometric parameters (dry leaf mass, the number of leaves, plant height) and SG parameters (the concentrations of rebaudioside A (RebA) and stevioside (Stev), total concentration of SGs (RebA+Stev), ratio RebA/Stev in stevia leaves); and (**B**) morphometric parameters (dry leaf mass, the number of leaves, plant height) and biochemical parameters (total phenolic content (TPC), total flavonoid content (TFC), antioxidant activity (AA) in stevia leaves).

The PCA was performed to assess the relationship and differences between measured parameters in the control and treatment groups by visualising the large set of multivariate data obtained from individual plants. The PCA biplots show dots representing samples belonging to different treatment groups and expressing multivariate information based on morphometric parameters (dry leaf mass, the number of leaves, plant height) and SG parameters (the concentrations of rebaudioside A (RebA) and stevioside (Stev), the total concentration of SGs (RebA+Stev), ratio RebA/Stev) (Figure 4A), and morphometric parameters (dry leaf mass, the number of leaves, plant height) and biochemical parameters (total phenolic content (TPC), total flavonoid content (TFC), antioxidant activity (AA) in stevia leaves) (Figure 4B) in stevia leaves.

In the case of morphometric data combined with data of biochemical parameters (TPC, TFC and AA) (Figure 4B), the biplot dots from different treatment groups are scattered and do not form clusters. Therefore, distinct differences between the control and CP2, CP5 and CP7 groups cannot be determined. In the case of morphometric data combined with SG data (Figure 4A), the biplot dots form overlapping clusters with some outliers. This set of data indicates distinct differences between the control and treatment groups, especially for a longer duration of CP treatment.

**Figure 5:**
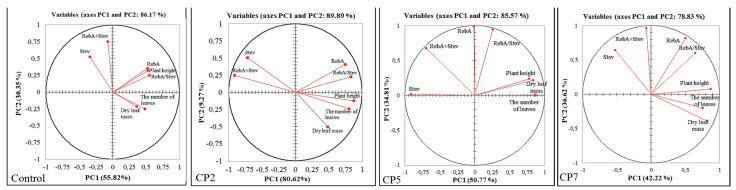
The correlation circles of the morphometric parameters (dry leaf mass, the number of leaves, plant height) and SG parameters (the concentrations of rebaudioside A (RebA) and stevioside (Stev), total concentration of SGs (RebA+Stev), ratio RebA/Stev in stevia leaves) of the control group and treatment groups (CP2, CP5 and CP7).

**Figure 6:**
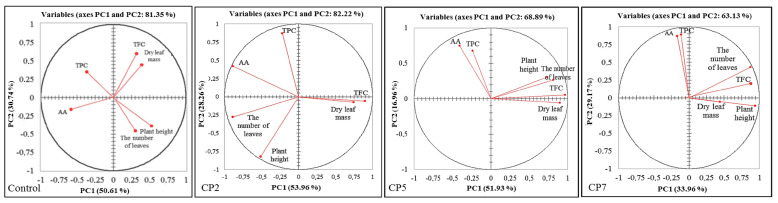
The correlation circles of the morphometric parameters (dry leaf mass, the number of leaves, plant height) and biochemical parameters (total phenolic content (TPC), total flavonoid content (TFC), antioxidant activity (AA) in stevia leaves) of the control group and treatment groups (CP2, CP5, and CP7).

In the correlation circles, each measured parameter was plotted against the first two of the principal components (PC1 and PC2) and parameters are shown as vectors for each treatment group (Figure 5 and Figure 6), representing the combined strength of the relationships between variables. The angle between two vectors gives the degree of correlation: two vectors with an angle < 90° show a positive correlation, and two vectors with an angle > 90° have a negative correlation. The correlation circles of the morphometric parameters and SG parameters (Figure 5) revealed that in the control group, plant height strongly positively correlated with RebA and RebA/Stev, and the number of leaves strongly positively correlated with leaf mass. All CP treatments weaken the positive correlation of plant height with RebA and RebA/Stev, and the correlation of number of leaves with leaf mass. A negative correlation of plant height with RebA+Stev is induced by CP2. A strong positive correlation of RebA with RebA/Stev, and a strong negative correlation of leaf mass with Stev were not influenced by CP treatment.

The correlation circles of the morphometric parameters and TPC, TFC and AA show similar patterns for CP5 and CP7 (Figure 6). Plant height and the number of leaves strongly negatively correlate with TPC in the control group, CP5 and CP7 treatment diminishes this correlation and induces a strong positive correlation of TPC with AA. A strong positive correlation of leaf mass with TFC was characteristic for all experimental groups.

In addition to PCA, the correlation was analysed by applying Pearson’s correlation coefficients between morphometric parameters and SG parameters, and morphometric parameters and other biochemical parameters of control and CP groups. Correlation matrices of Pearson’s correlation coefficients are shown in Tables S1–S8. A few morphometric parameters that can be used as markers (based on statistically significant Pearson’s correlation coefficients) for the prediction of biochemical content were determined: (1) the plant height positively correlates with RebA amount in the control group; (2) the leaf dry mass negatively correlates with RebA+Stev and AA in the CP2 group; (3) the number of leaves negatively correlates with Stev and RebA+Stev, and the leaf dry mass positively correlates with TFC in the CP5 group; (4) the leaf dry mass negatively correlates with Stev in the CP7 group.

## 3. Discussion

It has been demonstrated earlier that seed treatment with CP results in increased amounts of different metabolites in various plants: vitamin C, caffeic acid derivatives in purple coneflower (*Echinacea purpurea*) [24], non-psychotropic cannabinoids in industrial hemp (*Cannabis sativa*) [25], isoflavones in red clover (*Trifolium pratense*) [26,27] and different secondary metabolites in common buckwheat (*Fagopyrum esculentum*) [28]. We have recently reported for the first time that short-time pre-sowing treatment of stevia seeds with low-pressure CP can be a powerful tool for the enhancement of biosynthesis/accumulation of RebA and Stev, but CP treatments decreased the RebA/Stev ratio, the content of phenolics, flavonoids and antioxidant activity [29]. However, a different cultivar and different type of CP device (DBD) were used in this study, therefore a direct comparison of the CP-induced effects should be made with caution.

We did not obtain stimulation of germination using pre-sowing seed treatment with DBD CP which was characteristic of seeds treated with low-pressure CP [29]. The absence of stimulation can be explained by different treatment modes, cultivars and germination conditions (in vitro and in soil) used in these studies. In addition, the averaged morphometric parameters of seedlings were also not affected by seed treatment with CP. Thus, the results of this study represent the evidence that plant response to seed exposure to CP may proceed without observable changes in seedling emergence and growth; instead, it can be manifested only by changes in the content of a certain set of secondary metabolites, such as steviol glycosides.

The potential of pre-sowing seed treatment with CP to stimulate the production of main steviol glycosides—RebA and Stev—in *Stevia rebaudiana* plants in this study was different compared to our previous study [29]. As it was mentioned before, it may be due to the different plasma sources or cultivars. RebA was increased up to 7.1-fold in [29], but in this study, it was only by a maximum of 2.5-fold. RebA lacks the liquorice off-taste and lingering sweet aftertaste, which is characteristic of Stev. Therefore, in the stevia product industry, the stimulation of RebA biosynthesis rather than Stev and a higher RebA/Stev ratio is preferable. The aftertaste is eliminated if RebA and Stev are present at least in equal quantities [32]. CP2 induced the increase in the RebA/Stev ratio from 0.34 to 0.89 in this study, meaning that the taste quality was increased. This effect was opposite to the effect obtained in our previous study [29] where CP drastically decreased the RebA/Stev ratio. A preliminary hypothesis that CP decreases the RebA/Stev ratio in RebA-rich cultivars (where RebA is more abundant than Stev) such as Criolla, and increases it in low-RebA cultivars can be formulated for future studies.

The question of whether the yield of steviol glycosides per plant or cultivation area is still higher when plants follow the tendency to be smaller in CP-treated groups is important from the economical side of view. Although the decrease in plant yield (leaf dry mass per plant) is not statistically significant and the increase in SG amounts after all CP treatments is, the calculated total SGs amount per plant is significantly higher compared to the control (*p* ˂ 0.05) by 49% in the CP5 group only. In the CP2 and CP7 groups, it is higher by 20% and 19%, respectively compared to the control; however, this difference is statistically insignificant. For more precise economic benefit evaluation, the SG yield should be calculated over several seasons in a larger scale experiment with natural growing conditions in the field with occurring abiotic and biotic stress, incorporating equipment and energy expenses into the calculation. CP may have an additional complex effect on plant adaptivity and biomass gain in natural conditions.

Despite numerous attempts to elucidate the molecular mechanisms underlying the effects of seed exposure to cold plasma, the exact changes in biochemical pathways remain elusive. The reactive oxygen and nitrogen species (RONS) formed in plasma are involved in a variety of seed processes, including maturation, ageing, and germination, followed by seedling growth [33,34]; however, the uptake of RONS from plasma by seeds was not detected until recently. RONS introduction to seeds by similar DBD plasma as used in this study was recently demonstrated by Okumura et al. [35]. The study provided experimental evidence obtained by LC-MS that nitrate ion NO_3_^−^ is introduced in lettuce seeds as RONS upon irradiation with atmospheric pressure air DBD plasma. The feasibility of NO_3_^−^ introduction in seeds by air DBD plasma irradiation at atmospheric pressure was assessed using 1D simulation. The putative reactive species participating in NO_3_^−^ generation were NO, NO_2_, NO_2_^−^, NO_3_, HNO_3_, HNO_2_ and N_2_O_5_ [35]. The action of NO_3_^−^ in seeds and plants is very complex and results from its dual function as a nutrient and a signal [36]. Seed priming with NO_3_^−^ not only stimulates germination (reviewed in [36]) but can further induce biomass accumulation in the sprouts, and increase content of total mineral nutrients, pigments, vitamins, essential amino acids and some secondary metabolites [37]. The NO_3_^−^ ability to increase SG concentration in stevia leaves without influencing leaf biomass formation was demonstrated by Sun et al. [38]. The transcriptomic analysis revealed that such effects are likely due to the upregulation of the terpenoid synthesis pathway and that this NO_3_^−^-induced change could be attributed to the transcription factors belonging to MYB and/or WRKY families. These data imply that NO_3_^−^ may be one of the crucial plasma components responsible for SG synthesis stimulation in stevia.

In contrast to CP-induced stimulation in SG production, DBD CP treatment did not affect the total phenolic content (TPC), flavonoid content (TFC) or antioxidant activity. These values were strongly decreased after low-pressure CP treatment [29]. These findings show that CP does not induce the increase of these compounds, and consequently antioxidant activity, and may even inhibit their accumulation in stevia plants.

Stevia is a naturally cross-pollinated species; therefore, open-pollinated cultivars and cultigens are heterogeneous [39]. Therefore, the correlations of morphological traits which serve as important agronomic and economic traits with SGs and other bioactive secondary metabolites are useful for cultigen characterisation. For CP applications on stevia seeds for the improvement of plant biochemical composition, the correlations between different traits are of high predictive importance. Additionally, the positive effect on SGs and other metabolites should be balanced with economic traits (yield, disease resistance, etc.). These complex effects of CP in stevia have not been investigated. Some associations between traits are already determined in various cultigens, but the results are contradictory. RebA was negatively correlated with Stev [40,41] and dry leaf mass [42], but positively correlated with yield [41]. Some studies showed that the yield was also positively correlated with Stev [42], while other studies showed this correlation was negative [41,43]. We have determined (Figure 5) that in untreated (control) stevia plants, plant height strongly positively correlates with RebA and RebA/Stev, and the number of leaves strongly positively correlates with leaf mass. All CP treatments weaken the positive correlation of plant height with RebA and RebA/Stev, and the correlation of the number of leaves with leaf mass. A negative correlation of plant height with RebA+Stev was induced by CP2. A strong positive correlation of RebA with RebA/Stev, and a strong negative correlation of leaf mass with Stev were not influenced by CP treatment.

Next to diterpenes SGs, the non-sweetener fraction is rich in phenolic compounds, giving additional health benefits to the leaf material and adding extra value to the product. We have shown that plant height and number of leaves is strongly negatively correlated with TPC in the control group. CP5 and CP7 treatments diminish this correlation and induce a strong positive correlation of TPC with AA. That may indicate possible CP-induced changes in TPC composition resulting in different capacity to scavenge DPPH. A strong positive correlation of leaf mass with TFC was characteristic for all experimental groups.

**Figure 7:**
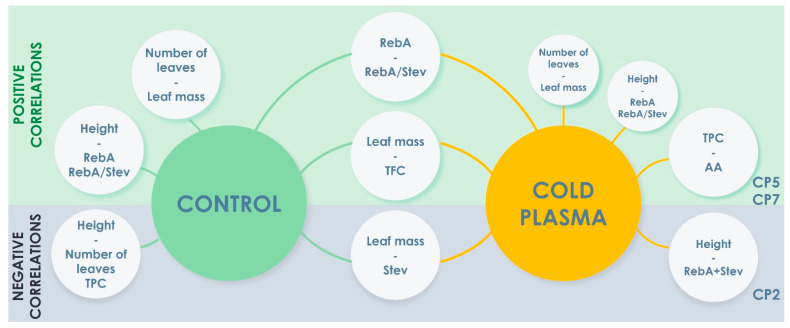
Schematic overview of CP-induced changes in correlations of variables deduced from PCA analysis. Smaller circles denote weaker correlations compared to control.

The main effect of pre-sowing seed treatment with CP in this study was SG production stimulation. This treatment did not affect the mean values of TPC, TFC or AA and has a general tendency to decrease leaf dry mass per plant, number of leaves and plant height. A summarised schematic overview of CP-induced changes in variable correlations is presented in Figure 7. The upper panel of the figure shows positive correlations, the lower panel shows the negative correlations, the side circles show unique relationships for control or CP groups and the central circles show constant correlations which stay unchanged after CP treatment. This analysis of individual plants revealed some possible diagnostic morphometric traits that can be used to predict chemical composition changes in stevia plants after pre-sowing seed treatment with CP (Figure 7): (1) CP-induced tendency of decreased plant height may result in a higher total amount of SGs when a short treatment of 2 min is applied; (2) the lower plant height may result in lower RebA and RebA/Stev. which is also an even stronger characteristic of the control group. No other correlations between different parameter groups (morphometric parameters–secondary metabolites) induced by CP were observed. Correlation matrices of Pearson’s correlation coefficients reveal that the plant height negatively correlates with RebA+Stev in the CP2 group, the number of leaves negatively correlates with Stev and RebA+Stev in the CP5 group and the leaf dry mass negatively correlates with Stev in the CP7 group. In the control group, as both PCA and Pearson’s correlation analyses show, the plant height positively correlates with RebA amount. The same correlation was previously demonstrated in a soil salinity effect study [44] and many other seed priming studies. Since no research on CP application on stevia seeds has been performed so far, CP-induced effects can only be compared to the correlations obtained in studies of stevia reaction to different environmental stressors. Like CP2-induced results, a negative correlation of plant height with RebA+Stev was observed in water deficit stress conditions [45]. The negative correlation of the number of leaves with Stev and RebA+Stev as observed in the CP5 group was also shown in stevia responding to moderate water supply restrictions [46]. Leaf dry mass negative correlation with Stev as observed in the CP7 group was also demonstrated in the study characterising 16 stevia cultigens [41]. The CP-induced decrease in at least one morphometric parameter lessens the overall effect of stimulation of SG biosynthesis. The goal to be achieved by any agricultural technology is an increased, or at least not reduced, biomass of high quality. Therefore, further tuning of CP parameters, dosage, growing conditions and selection of a suitable cultivar are needed to maximise the biomass and the yield of secondary metabolites in stevia.

## 4. Materials and Methods

### 4.1. Chemicals and Reagents

The standard of rutin, galic acid, Folin–Ciocalteu’s phenol reagent, HPLC grade methanol, 2,2-diphenyl-1-picrylhydrazyl and ethanol were obtained from Sigma Aldrich (St. Louis, MO, USA), stevioside and rebaudioside A were from TransMIT (Geiben, Germany), HPLC-grade acetonitrile and sodium acetate were from Sharlau Chemie S. A. (Sentmenat, Spain), HCl, sodium carbonate and acetic acid were from Carl Roth (Karlsruhe, Germany), hexamethylenetetramine and aluminium chloride were from Thermo Fisher Scientific (Lancashire, UK). All solutions were prepared with ultrapure 18.2 MΩ water from a Watek ultrapure water purification system (Watek Ltd., Ledeč nad Sázavou, Czech Republic).

### 4.2. Plant Material

Seeds of *Stevia rebaudiana* Bertoni were obtained from the commercial seed source company “Agrofirma SĖKLOS, UAB” (Vilnius, Lithuania). Seeds were sealed in paper bags and stored in a refrigerator (+4 °C) under dry conditions in the dark until the experiment date when they were irradiated with dielectric barrier discharge CP.

### 4.3. Seed Treatment with CP

**Figure 8:**
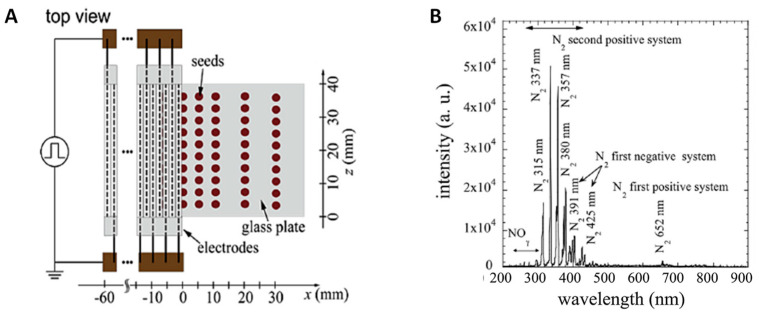
Schematic diagram of atmospheric pressure DBD device (**A**) and optical emission spectrum of air plasma (**B**).

Plasma irradiation of seeds was carried out by a scalable dielectric barrier discharge (DBD) device, described in more detail earlier [47,48,49] and shown in Figure 8A. For the device, 20 rods 2 mm in diameter and 60 mm long were placed as a planar shape with spacing of 0.2 mm. The rod electrode consisted of a stainless steel rod of 1 mm in diameter and a ceramic tube of 1 mm in inner diameter and of 2 mm in outer diameter. One layer of seeds (100 seeds) was placed under the electrode on the glass tray so that the distance from electrode and seed surface was 8 mm. Discharges were produced in the air between the gap of the electrodes by applying pulsed voltage of 7 kV (LHV-09K, Logy Electric Co. Ltd., Tokyo, Japan). The repetition frequency and the discharge power density were 14.4 kHz and 3.05 W/cm^2^, respectively, the total power was 4.64 W and the area of the plasma 1.52 cm^2^. The optical emission spectrum of plasma is shown in Figure 8B. Seeds were treated with DBD plasma at room temperature, and relative humidity of the air was kept at 50 ± 5% by applying an ultrasonic humidifier in the closed chamber where DBD plasma electrodes were placed. Based on some our previous studies on different plant seeds [26,27,29,50,51], the chosen duration for seed treatments was 2, 5 and 7 min (this treatment is further abbreviated as CP2, CP5 and CP7, respectively).

### 4.4. Seed Germination Test and Plant Cultivation

The untreated (control) seeds and CP-treated seeds were evenly distributed on the humidified substrate surface (4 growth containers (9 × 9 × 10 cm) of 25 seeds each) and watered with 50 mL distilled water. Growth containers were filled with substrate BioSoil (SIA “Green-PIK LAT”, reg. No. K0.02-1386-16, Lauces, Latvia), consisting of high-quality vermicompost, moss peat and sand. Characteristics: nitrogen (N)—0.3%, phosphorus (P_2_O_5_)—0.2%, potassium (K_2_O)—0.3%, organic substances—min 14.6%, humidity—max 30%, pH 6–7. Growth containers with seeds were placed in a climatic chamber (KK 750, Pol-Eko sp. k., Wodzisław Śląski, Poland) with automatic control of relative humidity (60%), light and temperature. Alternating light regime was maintained in the chamber (16 h light, 8 h dark) and constant temperature of 25 ± 1 °C. Seeds were provided additional water, if necessary, to prevent drying. A seed was considered germinated when the initial emergence of the radicle was observed. Germinated seeds were counted daily until their number stopped increasing.

The effect of CP on germination was estimated by the induced changes in parameters of germination kinetics, derived using application of Richards’ function [30] for the analysis of germinating seed population [31]: Vi (%)—the final germination percentage indicating seed viability, Me (days)—the median germination time (t_50%_) indicating the germination halftime of a seed lot or germination rate, and Qu (days)—the quartile deviation indicating the dispersion of germination time in a seed lot (half of seeds with an average growth time germinate in the range Me ± Qu).

After germination, seedlings were grown for 12 weeks under greenhouse conditions, with a long day photoperiod (16 h light, 8 h dark), relative humidity of 60% and constant temperature of 25 ± 1 °C.

### 4.5. Morphometric Measurements

The plant height, leaf number and leaf dry mass per plant (leaf mass) were measured in individual 12-week-old plants (n = 7).

### 4.6. Plant Material Preparation for Extraction

The leaves were collected from 12-week-old plants and dried at 40 °C for 24 h. Dried leaves were powdered using a batch mill with disposable grinding chamber (Tube-Mill control, IKA, Staufen, Germany) and stored at room temperature.

### 4.7. Extract Preparation for Steviol Glycosides (SGs) Analysis

The leaf powder was mixed with 70% ethanol at the ratio 1:50 (*w/v*). The extraction was carried out in triplicate by sonication for 60 min at 25 °C. The mixture was centrifuged at 16,000× *g* for 10 min, the supernatant was collected and kept at −20 °C until analysis.

### 4.8. Extract Preparation for the Analysis of Total Phenolic, Flavonoids Content and Antioxidant Activity

The leaf powder was mixed with 85% methanol at the ratio 1:5 (*w/v*). The extraction was carried out in triplicate by sonication for 15 min at 25 °C. The mixture was centrifuged at 16,000× *g* for 10 min, the supernatant was collected and kept at −20 °C until analysis.

### 4.9. HPLC Analysis of Steviol Glycosides

Steviol glycosides rebaudioside A (RebA) and stevioside (Stev) were separated and quantified using high-performance liquid chromatography (HPLC) [52]. An Agilent 1200 series HPLC system (Agilent Technologies Inc., Santa Clara, CA, USA) with a diode array detector was used. Samples were filtered through a syringe filter with a PVDF membrane (pore diameter 0.22 µm) and separated on a reversed phase column (Purospher STAR RP-18e 5 µm Hibar 2 × 250 mm, Merck, Germany) with a precolumn. Injection volume was 10 µL at 70 °C column temperature. Isocratic elution at a flow rate of 0.25 mL min^−1^ with a mobile phase consisting of 70% deionised water acidified with HCl to pH 2.75 and 30% acetonitrile was used for separation with an additional washing step with 50% acetonitrile. RebA and Stev were detected at the wavelength of 210 nm. Calibration was performed by plotting the peak area responses against the concentration values in the concentration range from 1 to 1000 µg mL^−1^ with linear dependence for both analytes. Each analysis was repeated three times, and the mean value was used.

### 4.10. Determination of Total Phenolic Content

Total phenolic content was determined using the modified Folin–Ciocalteu method [52]. First, 0.2 mL of stevia extract was mixed with 1 mL of 0.2 N Folin–Ciocalteu reagent and 0.8 mL 7.5% sodium carbonate solution. After 60 min of incubation in the dark at room temperature, absorbance was measured at 760 nm. Gallic acid was used as a standard, and results were expressed by mg of gallic acid equivalent (GAE) mg g^−1^ of dry weight (DW).

### 4.11. Determination of Total Flavonoid Content

Total flavonoid content was analysed using a colorimetric method based on the complexation of phenolic compounds with Al (III) [52]. First, 80 µL of stevia extract was mixed with 1920 µL of a reagent containing 40% ethanol, 0.7% acetic acid, 0.4% hexamethylenetetramine and 0.6% aluminium chloride. After 30 min of incubation in the dark at 4 °C, absorbance was measured at 407 nm. Rutin was used as a standard, and results were expressed by mg of rutin equivalent (RUE) mg g^−1^ of DW.

### 4.12. Determination of Antioxidant Activity

Antioxidant activity was measured based on the scavenging of the stable 2,2-diphenyl-1-picrylhydrazyl free radical (DPPH) as described [52]. Accordingly, 50 µL of stevia extract were mixed with 1950 µL of a DPPH solution (0.025 mg mL, prepared in acetonitrile:methanol:sodium acetate buffer (100 mM, pH 5.5) (1:1:2)). After 15 min of incubation in the dark at room temperature, absorbance was measured at 515 nm. Rutin was used as a standard, and antioxidant activity was expressed by mg of rutin equivalent (RUE) mg g^−1^ of DW.

### 4.13. Statistical Analysis

All measurements of various parameters between the control and pre-sowing treatment groups were expressed as mean ± SEM and compared using Student’s *t*-tests for unpaired samples. Statistical significance of CP effects was assumed to be statistically significant when *p* < 0.05. Principal component analysis (PCA) was performed using XLSTAT Software (XLSTAT, 2022, New York, NY, USA) to analyse the association between morphometric and biochemical properties of stevia grown from seeds exposed to CP treatment of different durations, and control seeds, and represent differences across individuals within given data.

## Figures and Tables

**Table 1:** Indices of germination kinetics of *Stevia rebaudiana* seeds derived from Richards plots.

Group	Vi, %	Me, Days	Qu, Days
Control	40.0 ± 5.0	3.9 ± 0.1	0.16 ± 0.01
CP2	36.0 ± 5.4	4.0 ± 0.2	0.64 ± 0.10 *
CP5	44.0 ± 4.0	4.0 ± 0.1	0.23 ± 0.02 *
CP7	36.0 ± 4.0 *	3.8 ± 0.2	0.21 ± 0.02 *

Vi, the final germination percentage; Me, the median germination time; Qu, the quartile deviation; Means ± SEM are presented (n = 4); * statistically significant difference compared to control (*p* < 0.05).

**Table 2:** Morphometric parameters of *Stevia rebaudiana* plants grown in the substrate for 12 weeks from control and CP-treated seeds.

Group	Leaf Dry Mass per Plant, g	Number of Leaves	Plant Height, cm
Control	1.46 ± 0.12	29.60 ± 0.98	27.20 ± 3.04
CP2	1.44 ± 0.18	27.33 ± 2.91	24.00 ± 3.18
CP5	1.18 ± 0.12	26.57 ± 0.72	22.50 ± 2.14
CP7	1.23 ± 0.07	27.60 ± 1.17	22.20 ± 2.54

Means ± SEM are presented (n = 7)

**Table 3:** Effect of CP on *Stevia rebaudiana* leaf steviol glycoside content (mg·g^−1^ of DW) and ratio.

	RebA	Stev	RebA+Stev	RebA/Stev	RebA/(RebA+Stev)	Stev/(RebA+Stev)
Control	42.72 ± 12.73	136.56 ± 20.92	179.28 ± 21.15	0.34 ± 0.11	0.24 ± 0.06	0.76 ± 0.06
CP2	97.73 ± 8.06 *	120.75 ± 20.90	218.48 ± 14.39 *	0.89 ± 0.23 *	0.45 ± 0.06 *	0.55 ± 0.06 *
CP5	108.56 ± 23.96 *	222.06 ± 22.26 *	330.62 ± 17.78 *	0.56 ± 0.14	0.32 ± 0.06	0.68 ± 0.06
CP7	79.55 ± 20.59 *	174.45 ± 20.47 *	253.99 ± 29.01 *	0.48 ± 0.14	0.30 ± 0.06	0.70 ± 0.06

Means ± SEM are presented (n = 7); *, statistically significant difference compared to control (*p* < 0.05).

**Table 4:** The content of total phenolic compounds (TPC), total flavonoid compounds (TFC) and antioxidant activity (AA) in leaves of *Stevia rebaudiana* leaf grown for 12 weeks from control and CP-treated seeds.

	TPC (mg GAE g^−1^ DW)	TFC (mg RUE g^−1^ DW)	AA (mg RUE g^−1^ DW)
Control	81.07 ± 9.37	56.17 ± 2.14	32.15 ± 0.93
CP2	71.41 ± 2.03	57.92 ± 6.35	30.10 ± 2.06
CP5	83.29 ± 7.44	49.06 ± 2.92	27.17 ± 3.84
CP7	74.11 ± 4.84 *	53.25 ± 3.13	30.29 ± 2.25

GAE, gallic acid equivalents; RUE, rutin equivalents; Means ± SEM are presented (n = 7); *, statistically significant difference compared to control (*p* < 0.05).

## Data Availability

The data presented in this study are available on request from the corresponding author. The data are not publicly available due to the patent application process.

## Supplementary Materials

The following supporting information can be downloaded at https://www.mdpi.com/article/10.3390/plants12081585/s1, Table S1: Correlation matrix showing Pearson’s correlation coefficients for morphometric parameters (dry leaf mass, the number of leaves, plant height) and SGs parameters (the concentrations of rebaudioside A (RebA) and stevioside (Stev), total concentration of SGs (RebA+Stev), ratio RebA/Stev in stevia leaves) of the control group; Table S2: Correlation matrix showing Pearson’s correlation coefficients for morphometric parameters (dry leaf mass, the number of leaves, plant height) and biochemical parameters (total phenolic content (TPC), total flavonoid content (TFC), antioxidant activity (AA) in stevia leaves) of the control group; Table S3: Correlation matrix showing Pearson’s correlation coefficients for morphometric parameters (dry leaf mass, the number of leaves, plant height) and SGs parameters (the concentrations of rebaudioside A (RebA) and stevioside (Stev), total concentration of SGs (RebA+Stev), ratio RebA/Stev in stevia leaves) of CP2 group; Table S4: Correlation matrix showing Pearson’s correlation coefficients for morphometric parameters (dry leaf mass, the number of leaves, plant height) and biochemical parameters (total phenolic content (TPC), total flavonoid content (TFC), antioxidant activity (AA) in stevia leaves) of CP2 group; Table S5: Correlation matrix showing Pearson’s correlation coefficients for morphometric parameters (dry leaf mass, the number of leaves, plant height) and SGs parameters (the concentrations of rebaudioside A (RebA) and stevioside (Stev), total concentration of SGs (RebA+Stev), ratio RebA/Stev in stevia leaves) of CP5 group; Table S6: Correlation matrix showing Pearson’s correlation coefficients for morphometric parameters (dry leaf mass, the number of leaves, plant height) and biochemical parameters (total phenolic content (TPC), total flavonoid content (TFC), antioxidant activity (AA) in stevia leaves) of CP5 group; Table S7: Correlation matrix showing Pearson’s correlation coefficients for morphometric parameters (dry leaf mass, the number of leaves, plant height) and SGs parameters (the concentrations of rebaudioside A (RebA) and stevioside (Stev), total concentration of SGs (RebA+Stev), ratio RebA/Stev in stevia leaves) of CP7 group; Table S8: Correlation matrix showing Pearson’s correlation coefficients for morphometric parameters (dry leaf mass, the number of leaves, plant height) and biochemical parameters (total phenolic content (TPC), total flavonoid content (TFC), antioxidant activity (AA) in stevia leaves) of CP7 group.
