# Supplementary material for: Cold Plasma-Induced Changes in Stevia rebaudiana Morphometric and Biochemical Parameter Correlations
Source: Plants (Basel). 2023 Apr 8;12(8):1585. doi: 10.3390/plants12081585 (PMC10145628; doi:10.3390/plants12081585)
Supplement: Supplementary file 1 [file plants-12-01585-s001.zip › plants-2311156-supplementary.pdf]

Supplementary Material

Supplementary Tables

Table S1. Correlation matrix showing Pearson's correlation coefficients for morphometric parameters (dry leaf mass, the number of leaves, plant height) and SGs parameters (the concentrations of rebaudioside A (RebA) and stevioside (Stev), total concentration of SGs (RebA+Stev), ratio RebA/Stev in stevia leaves) of the control group.

Variables	RebA	Stev	RebA+Stev	RebA/Stev	Leaf dry mass per plant	Number of leaves
**Stev**	0.044					
**RebA+Stev**	0.319	0.817				
**RebA/Stev**	0.981*	-0.067	0.164			
**Leaf dry mass per plant**	0.409	-0.733	-0.285	0.524		
**Number of leaves**	0.533	-0.773	-0.444	0.563	0.242	
**Plant height**	0.955*	-0.011	0.263	0.912*	0.254	0.696*

Significant at 0.05 level of probability (*)

Table S2. Correlation matrix showing Pearson's correlation coefficients for morphometric parameters (dry leaf mass, the number of leaves, plant height) and biochemical parameters (total phenolic content (TPC), total flavonoid content (TFC), antioxidant activity (AA) in stevia leaves) of the control group.

Variables	TPC	TFC	AA	Leaf dry mass per plant	Number of leaves
**TFC**	-0.118				
**AA**	0.542	-0.725			
**Leaf dry mass per plant**	0.139	0.686	-0.715		
**Number of leaves**	-0.191	-0.343	-0.322	0.242	
**Plant height**	-0.802	0.067	-0.721	0.254	0.696

Significant at 0.05 level of probability (*)

Table S3. Correlation matrix showing Pearson's correlation coefficients for morphometric parameters (dry leaf mass, the number of leaves, plant height) and SGs parameters (the concentrations of rebaudioside A (RebA) and stevioside (Stev), total concentration of SGs (RebA+Stev), ratio RebA/Stev in stevia leaves) of CP2 group.

Variables	RebA	Stev	RebA+Stev	RebA/Stev	Leaf dry mass per plant	Number of leaves
**Stev**	-0.467					
**RebA+Stev**	-0.709	0.756				
**RebA/Stev**	0.822*	-0.611	-0.622			
**Leaf dry mass per plant**	0.089	-0.911	-0.633	0.244		
**Number of leaves**	0.422	-0.767	-0.978	0.927	0.644	
**Plant height**	0,544	-0,678	-0,948*	0,964	0,556	0,801*

Significant at 0.05 level of probability (*)

Table S4. Correlation matrix showing Pearson's correlation coefficients for morphometric parameters (dry leaf mass, the number of leaves, plant height) and biochemical parameters (total phenolic content (TPC), total flavonoid content (TFC), antioxidant activity (AA) in stevia leaves) of CP2 group.

Variables	TPC	TFC	AA	Leaf dry mass per plant	Number of leaves
**TFC**	-0.182				
**AA**	0.497	-0.833*			
**Leaf dry mass per plant**	-0.069	0.504	-0.725*		
**Number of leaves**	-0.105	-0.752*	0.611	-0.482	
**Plant height**	-0.500	-0.356	0.089	-0.252	0.759*

Significant at 0.05 level of probability (*)

Table S5. Correlation matrix showing Pearson's correlation coefficients for morphometric parameters (dry leaf mass, the number of leaves, plant height) and SGs parameters (the concentrations of rebaudioside A (RebA) and stevioside (Stev), total concentration of SGs (RebA+Stev), ratio RebA/Stev in stevia leaves) of CP5 group.

Variables	RebA	Stev	RebA+Stev	RebA/Stev	Leaf dry mass per plant	Number of leaves
**Stev**	-0.020					
**RebA+Stev**	0.655*	0.742*				
**RebA/Stev**	0.938*	-0.289	0.410			
**Leaf dry mass per plant**	0.128	-0.783	-0.678	0.108		
**Number of leaves**	0.011	-0.989*	-0.489*	0.167	0.833	
**Plant height**	0.188	-0.666	-0.378	0.361	0.758	0.811*

Significant at 0.05 level of probability (*)

Table S6. Correlation matrix showing Pearson's correlation coefficients for morphometric parameters (dry leaf mass, the number of leaves, plant height) and biochemical parameters (total phenolic content (TPC), total flavonoid content (TFC), antioxidant activity (AA) in stevia leaves) of CP5 group.

Variables	TPC	TFC	AA	Leaf dry mass per plant	Number of leaves
**TFC**	-0.335				
**AA**	0.284	0.000			
**Leaf dry mass per plant**	-0.035	0.581*	-0.493		
**Number of leaves**	-0.145	0.478	-0.117	0.555	
**Plant height**	-0.095	0.372	-0.100	0.508	0.672*

Significant at 0.05 level of probability (*)

Table S7. Correlation matrix showing Pearson's correlation coefficients for morphometric parameters (dry leaf mass, the number of leaves, plant height) and SGs parameters (the concentrations of rebaudioside A (RebA) and stevioside (Stev), total concentration of SGs (RebA+Stev), ratio RebA/Stev in stevia leaves) of CP7 group.

Variables	RebA	Stev	RebA+Stev	RebA/Stev	Leaf dry mass per plant	Number of leaves
**Stev**	0.110					
**RebA+Stev**	0.696	0.790*				
**RebA/Stev**	0.929*	-0.206	0.424			
**Leaf dry mass per plant**	0.079	-0.663*	-0.430	0.332		
**Number of leaves**	0.104	-0.243	-0.111	0.103	0.251	
**Plant height**	0.387	-0.234	0.070	0.464	0.309	0.751*

Significant at 0.05 level of probability (*)

Table S8. Correlation matrix showing Pearson's correlation coefficients for morphometric parameters (dry leaf mass, the number of leaves, plant height) and biochemical parameters (total phenolic content (TPC), total flavonoid content (TFC), antioxidant activity (AA) in stevia leaves) of CP7 group.

Variables	TPC	TFC	AA	Leaf dry mass per plant	Number of leaves
**TFC**	0.036				
**AA**	0.598	0.088			
**Leaf dry mass per plant**	-0.102	0.157	-0.226		
**Number of leaves**	0.207	0.313	0.274	0.251	
**Plant height**	-0.339	0.195	-0.132	0.309	0.789*

Significant at 0.05 level of probability (*)
